# The spatial specificity of sensory attenuation for self-touch

**DOI:** 10.1016/j.concog.2021.103135

**Published:** 2021-07

**Authors:** Franziska Knoetsch, Eckart Zimmermann

**Affiliations:** Institute for Experimental Psychology, Heinrich Heine University Düsseldorf, Universitätsstr. 1, 40225 Düsseldorf, Germany

**Keywords:** Sensory attenuation, Efference copy, Self-touch, Spatial sensitivity

## Abstract

Self-touch is sensed less intense as touch produced by another person. According to the standard explanation, if predicted and actual sensations match, the intensity of the touch will be reduced. Here, we asked whether sensory attenuation is spatially specific. To this end, participants laid their left hand under a metal arc on which a force sensor was mounted. Pressing the sensor caused one of two motors to rotate a lever that either touched the index or the ring finger. We took care that no cues would reveal in advance which of the motors would move in order to leave participants uninformed about the finger that will be stimulated. Any reduction in felt intensity of the touch could therefore be induced only by the efference copy of the touching movement. We found strong spatial specificity of sensory attenuation of self-touch, selecting between two fingers of the same hand.

## Highlights

• Sensory attenuation is spatially specific.
• Attenuation selects between neighboring fingers.
• Specificity is independent of purely perceptual prediction.

## 1 Introduction

The perception of self-produced sensory events is often illustrated by the famous question why we cannot tickle ourselves (for reviews, see Blakemore et al., 2000, Hughes et al., 2013, Dogge et al., 2019). Part of the reason why we cannot tickle ourselves is the predictability of the upcoming sensory event. However, predictability alone cannot be the sole reason as predicted touch produced by someone else will still feel ticklish. Thus, only touch that is generated by ourselves makes us lose the feeling of ticklishness.

Which signals cause sensory attenuation in self-touch to occur? In principle, any kind of prediction that a finger touches a body part might be sufficient to attenuate the corresponding sensation. Such a prediction might be delivered by a copy of the motor plan that generates the touching movement, i.e. an efference copy. If the efference copy of the pressing movement plays a role, attenuation should only be triggered by active but not by passive movements. Indeed, only when subjects actively pressed a pressure sensor that led to a touch of their arms, sensory attenuation occurred but not when the press was induced by a passive movement of the finger (Kilteni, Engeler, & Ehrsson, 2020). In a forward model architecture, the efference copy is used to predict the sensory consequences of the movement. The accuracy of the prediction will then modulate the intensity of the felt touch. The comparator model suggests that if the prediction error is small, the touch will be perceived as less intense as one resulting from an external movement (Blakemore, Wolpert, & Frith, 1998). One important feature that can dissociate between models of sensory attenuation is its spatial specificity. Spatial specificity has been investigated in previous research for tickliness ratings after subjects produced tactile stimuli by moving a robot arm with their left hand over their passive right hand (Blakemore, Frith, & Wolpert, 1999). Tactile stimuli felt less ticklish when trajectory perturbations were applied which varied the direction of the tactile stimulus movement relative to the movement of the left hand. Bays and Wolpert (2008) tested the force matching task and shifted the position of the touching finger from the passive finger that received the tactile stimulus. Sensory attenuation was significantly reduced when both fingers were separated 10 cm or more. Kilteni, Houborg, and Ehrsson (2019) asked subjects to touch their left hand with a tool that participants were holding in their right hand. In this setup, they found that force was matched veridically, i.e. sensory attenuation was absent at a distance of 15 cm or more.

Sensory attenuation as a consequence of self-touch is often discussed together with attenuation of external or environment-related events (Dogge, Custers, & Aarts, 2019). When a subject actively presses a button that produces a sound or a visual stimulus, the acoustic sensation appears attenuated (Dogge et al., 2019). Although in both phenomena, active movements induce an attenuation of a sensation, it is unclear if they are produced by the same mechanism. The learning history for self-touch is very different from the production of external events, where action-effect contingencies are rather arbitrary. The strongest argument for a dissociation of both phenomena is provided by the spatial specificity of self-touch. The spatial selectivity of sensory attenuation in self-touch (Bays and Wolpert, 2008, Kilteni et al., 2019) necessitates a mechanism that operates in a body-centered reference frame. Such a mechanism can hardly provide the attenuation of external events. Interestingly, body-related sensory attenuation can also be tested outside the self-touch paradigm. If subjects press a button that produces a tactile impulse on a finger of their resting hand, sensory attenuation likewise occurs (Burin, Pyasik, Salatino, & Pia, 2017). This experiment demonstrates that indeed the production of external events is different from self-touch: Sensory attenuation occurred although the finger that presses the button and the finger that receives the impulse were spatially separated.

Several models have been suggested to explain the sensory attenuation of self-touch. The comparator model idea does not provide an explanation on how the cancellation of the tactile sensory impulse might work. Roussel, Hughes, and Waszak (2013) offer a mechanism that accounts for the cancellation. This mechanism is derived from ideomotor theory, which assumes that the preparation of a motor movement consists in the preactivation of the sensory consequences of that movement. Preactivation would increase the mean level of neural activity, that represents the predicted sensory consequences, to a pedestal level, making discrimination of the signal activation more difficult than discrimination of the signal activation from the baseline level. The comparator model of sensory attenuation has been criticized for associating prediction errors with the intensity of percepts (Brown, Adams, Paress, Edwards, & Friston, 2013). In addition, prediction errors update predictions over the history of sensory events, they are not directly related to the current percept. The recent literature on serial dependencies demonstrates how predictions can modulate perception in the following trial (Fischer and Whitney, 2014, Cicchini et al., 2014, Cicchini et al., 2018, Fritsche et al., 2017). Similarly, errors encountered in sensorimotor tasks change performance and perception in upcoming events (Cont & Zimmermann, 2021). It is thus unusual that in the comparator model sensory attenuation occurs within the same trial in which the error is registered. Brown et al. (2013) suggested a third alternative account of sensory attenuation based on active inference which is derived from predictive coding. Active inference claims that around the time of movements sensory processing prioritizes the proprioceptive consequences of these movements, thus leading to sensory attenuation in other sensory channels. This interpretation is consistent with results of self-generated external events that have been found in the visual (Cardoso-Leite et al., 2010, Desantis et al., 2014, Hughes and Waszak, 2011, Mifsud et al., 2016, Yon and Press, 2017; but see: Schwarz, Pfister, Kluge, & Kunde, 2018) and the auditory (Baess et al., 2009, Weiss et al., 2011) domain. However, as described above, it is not likely that both phenomena are generated by the same mechanism.

In the present study, we aimed to determine the spatial specificity for sensory attenuation of self-touch. This question can be addressed with an apparatus (as described in the material and methods section) that allows to dissociate the position of the active, touching finger, from the position of the passive area that is touched.

A selective spatial specificity would suggest that efference copy information is used to modulate the tactile intensity of the touched area. If sensory attenuation is restricted to the goal location of a movement, by definition only an efference copy signal can provide the corresponding coordinates predictively.

## 2 Material and methods

### 2.1 Participants

Twenty-five participants were tested in the experiment (age 18 to 56). The average age was 25.83 with a standard deviation of 2.04. 14 were females, 2 participants were left-handed. All participants were recruited through the Heinrich-Heine University Düsseldorf and received either course credit or payment of 10€/hour besides of the authors and participants working in the lab. Experimental procedures were approved by the local ethics committee of the psychological department of the Heinrich-Heine University Düsseldorf. Written informed consent was obtained prior to each experiment in accordance with the declaration of Helsinki.

### 2.2 Procedure

**Fig. 1:**
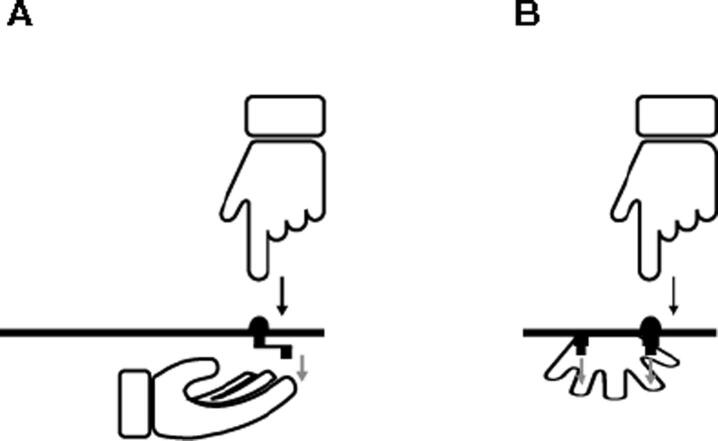
Experimental setup. The left hand was placed in an upside-down position underneath a metal arch on which the two motors were attached. The ring finger was placed underneath one motor, the index finger underneath the other. The sensor was on top of the metal arch, where the right hand rested. Once the sound occurred, the participant pushed the sensor with the right index finger which caused the motor to move the lever either on the index- or the ring finger. The gray arrows show the fingers that the levers touch.

In this study, participants placed their left arm on a support bar with their palm up and underneath a metal arc. Two motors (Savöx SC-1257 TG Motors) were mounted under the metal arc (see Fig. 1B). Each motor could move a lever that touched a finger with a controllable strength. Motors were driven by a micro-controller (Arduino nano) which was connected to a MacBook Pro. A custom-made Objective-C program controlled the timing of the motors by sending commands via the serial port of the micro-controller. The pressure sensor and the servo motor were connected to the micro-controller directly. Motor movement for probe rotations were controlled by the software uploaded on the micro-controller. Since information within the mircro-controller is processed in the micro seconds range, the delay between button press and motor rotation is mostly produced by the regulating time of the motor. The Savöx SC-1257 TG when used at 4.8 V (in our study 5 V was used) has a regulating time of 90 ms/60°. We let the motors rotate the connected lever by 20°. There was thus an approximate delay between button press and lever rotation of 30 ms.

Participants placed the left index finger and the left ring finger each underneath one of the motors, approximately 6 cm apart. Bubble wrap was additionally used to keep the left forearm in a comfortable position. In the Experiment, the right hand was placed on top of the metal arc where the index finger rested above the force sensor (FSR®, Interlink Electronics, Inc, Camarillo, CA 93012, USA). The right hand continuously rested above the force sensor by laying it on the metal arch. Participants were offered a pillow to rest their right elbow on in case the position was not comfortable enough. This way, participants only had to move their right index finger in order to push the force sensor to imply movement.

Thousand ms after a trial starts, an auditory cue appeared (Apple system sound) which indicated that the participant ought to press the force sensor with their right index finger. Once the sensor detected a press, one of the two motors rotated the lever in order to touch the respective finger. The rotation occurred either simultaneously with the press or 500 ms later. The time of rotation was randomized across trials. After the first rotation of the motor, a second rotation by the same motor occurred 700 ms later on the same finger where the first rotation occurred. Which motor moved (touching either the index or the ring finger) was randomized across trials. Participants could not see nor hear which motor moved. The strength of the first touch by the lever was kept constant across all trials (2 N). The strength of the second touch by the lever in a given trial was chosen randomly out of 7 possible reference magnitudes (0.5–3.5 N in 7 equidistant and equiprobable steps). Each of these 7 reference magnitudes was tested 10 times with presentations randomized across trials. The task of the participants was to decide which touch by the lever was stronger, the first or the second. They responded on a foot pedal (ECS-PPD-FP USB Dual function foot pedal) with their left or right foot. As soon as they responded, the next trial started.

In total, there were 2 presentation delays (0 ms or 500 ms touch relative to press on force sensor) times 2 fingers (index or ring finger) with 70 trials in each of these 4 conditions.

### 2.3 Data analysis

The Experiment had a 2x2 repeated measures within subject design with the factors finger (index or ring finger) and delay (0 ms or 500 ms). For the statistical analysis, IBM SPSS Statistics Software (Version 27) was used. In order to determine the perceived intensity of the first touch, we estimated a psychometric function for each observer for each condition. We averaged the responses for each of the 7 reference magnitudes and fitted a cumulative gaussian function to these data. We chose the mean parameter as the point of subjective equality that reflected the perceived intensity of the first touch.

## 3 Results

**Fig. 2:**
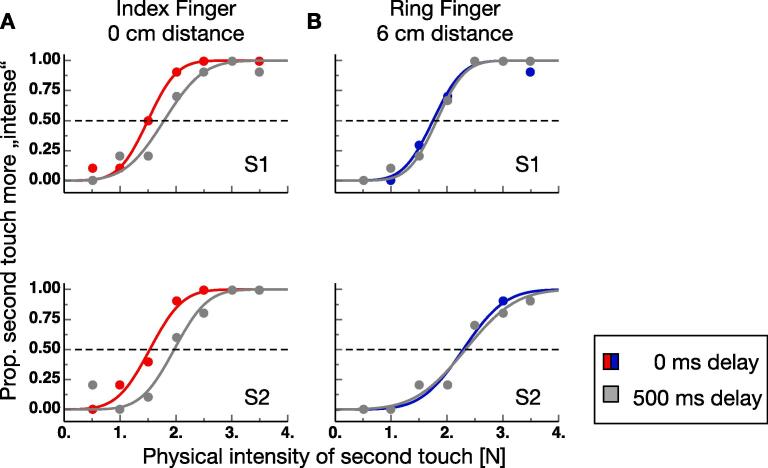
**A, B** Example psychometric functions from trials in which the touch by the lever occurred simultaneously with the press (colored points) and from trials in which it occurred 500 ms later (gray points). Stimulation on the index finger is shown in **A** and stimulation on the ring finger in **B**.

**Fig. 3:**
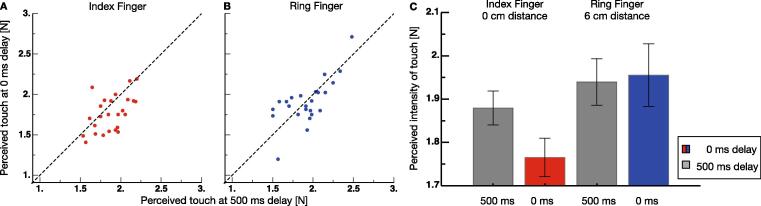
**A** Perceived touch intensity at 0 ms delay against perceived touch intensity at 0 ms delay measured at the index finger for all subjects. **B** Perceived touch intensity at 0 ms delay against perceived touch intensity at 0 ms delay measured at the ring finger for all subjects. **A** Average perceived intensity of the touch on the index and the ring finger for the two delays. Same color code as in Fig. 2. Error bars represent S.E.M.

Participants had to press the pressure sensor with their right index finger. The average press strength was 2.75 (S.E.M. 16.70) N. Fig. 2A shows example psychometric functions from two observers from trials in which the index finger was stimulated by the lever. Data from trials in which the lever touched the finger simultaneously with the press on the sensor are shown in red and data from trials in which the lever touch was 500 ms delayed are shown in gray. For both observers, psychometric functions in the 0 ms delay condition are shifted toward smaller intensities of the lever touch, indicating that they perceived the touch as less intense when it occurred simultaneous with their press versus when it occurred 500 ms later. This finding is a clear result of sensory attenuation. No indication of sensory attenuation was found for the two observers when they pressed the sensor - that was mounted above the index finger - and the ring finger was touched (see Fig. 2B). Psychometric functions were virtually identical for the 0 ms and the 500 ms delay conditions. This finding suggests that sensory attenuation is spatially selective for the goal location of the touching movement of their right hand. Fig. 3A, B show perceived touch intensity at 0 ms delay against perceived touch intensity at 500 ms delay for all subjects measured at the index and the ring finger. One can see that data measured at the ring finger cluster around the identity line (dashed line), which indicates similar perceived intensities at both delays. However, data measured at the index finger are shifted towards smaller perceived intensities at 0 ms delay.

Fig. 3C shows average perceived intensity of the lever touch for all conditions. As was seen for the single observers, a reduced apparent intensity could be measured for touches on the index finger when the touch occurred simultaneously with the press. On the ring finger, perceived touch intensity was nearly identical for touches that occurred simultaneously with or 500 ms delayed to the press on the sensor. After checking normality of the data with a Kolmogorov-Smirnoff test and variance homogeneity with a Levene test, we analyzed points of subjective equality between the first and second touch of the lever that were derived from the estimates of the psychometric functions. A 2x2 repeated measures ANOVA with the factors finger (index or ring finger) and delay (0 ms or 500 ms) revealed a significant main effect for the factor finger F(1,24) = 5.310, p = .03), confirming that sensory attenuation took place only on the index finger, i.e. the finger under the sensor. No significant effect for delay F(1,24) = 2.170, p = .154) was found. The significant interaction effect F(1,24) = 4.388, p = .047) revealed that sensory attenuation on the index finger was significantly stronger than on the ring finger. We followed up the significant interaction effect with Bonferroni corrected post-hoc tests and found a significant difference between the 0 ms and 500 ms delayed stimulation when tested at the index finger (p = .009, effect size: d = 0.55) and a significant difference between the index and the ring finger for stimulation applied at 0 ms (p = .018, effect size: d = 0.63).

These results demonstrate that sensory attenuation spatially selects the finger that is closest to the sensor which participants pressed. No significant differences for JNDs, neither for the factor finger (F(1,24) = 0.332, p = .57), nor for then factor delay (F(1,24) = 0.014, p = .91), nor for the interaction (F(1,24) = 0.042, p = .84) effect were found.

## 4 Discussion

In this study, we found that sensory attenuation for self-touch is spatially specific to the goal location of the touching finger. Participants had to press a button that induced a tactile impulse either on the finger directly underneath a button (index finger) or on a finger that was close to the button (ring finger). For both fingers, the presentation of the tactile impulse was either simultaneous with the button press or it occurred 500 ms after the button press. When comparing both presentation times for each finger, we found a significant reduction in perceived tactile intensity for the finger underneath the button when the tactile impulse occurred simultaneously with the button press. This difference was significantly stronger than that for the finger that was only close by, suggesting that sensory attenuation for self-touch is fine-tuned to the location of the touching finger.

Our results are consistent with previous findings reported in the literature on sensory attenuation. Blakemore et al. (1999) asked subjects to move a robot manipulator controlling a secondary device that stimulated their right hand. This self-produced condition yielded reduced tickle ratings compared to a passive condition in which only the robot controlled the movement of stimulation. When the experimenters applied trajectory perturbations that changed the direction of the stimulation relative to the movements of the subject, the tactile experience was rated as more ticklish. This result indicated that the sensory attenuation magnitude depends on how well the produced movement and its sensory effects match in direction. However, whereas Blakemore et al. (1999) demonstrated the effects of motion similarity on subjective ticklishness, we report the spatial specificity of sensory attenuation for neighboring fingers, objectively measured in a tactile intensity comparison task. Bays and Wolpert (2008) used a self-touch device and found a reduction of sensory attenuation when the touching and the touched finger were separated by 10 cm or more. Importantly, conditions in their study were tested blockwise. It has been reported recently that subjects adapt to the repeated exposure to a temporal delay between a touch and its effects (Kilteni, Houborg, & Ehrsson, 2019). A similar adaptation might occur for a spatial separation between the touch and its effects. Thus, in the experimental protocol used by Bays and Wolpert (2008), subjects might have adapted in the 10 cm separation condition. This might explain why we found a much tighter spatial specificity, even distinguishing neighboring fingers. Kilteni and Ehrsson (2017) instructed participants to press a force sensory with the left hand in order to apply a force to the right hand. They showed that sensory attenuation for the intensity of self-touch was reduced when a distance of 15 cm or more was introduced between both hands. In their study, the spatial separation conditions were tested in randomized order. However, in this study subjects could see the separation between pressing and touched finger.

The data of the present study show - in agreement with previous research (Kilteni and Ehrsson, 2017, Bays and Wolpert, 2008) - that sensory attenuation only occurs if there is a spatial match between the pressing and the touched finger. There are three possible signals available that code the position of the pressing finger: vision, proprioception and the efference copy of the motor plan. Recent research has demonstrated that only active movements generate sensory attenuation (Kilteni et al., 2020). As vision and proprioception were identical and only the availability of an efference copy varied between conditions in that study, vision and proprioception can be ruled out as relevant signals coding the position of the pressing finger in sensory attenuation.

For the touched finger, since it is resting, there are only two possible signals to retrieve its position: vision and proprioception. In previous research, subjects could see if their touch made contact with the resting finger (Kilteni and Ehrsson, 2017, Bays and Wolpert, 2008). In contrast, in our study subjects could not see their resting hand, nor could they see which motor lever applied the touch impulse. Only proprioception of the resting hand could reveal which finger was pressed. This signal might have been compared to the efference copy of the pressing finger movement. Only in case of a tight spatial match, sensory attenuation occurred. Alternatively, subjects might have known that their left index finger was closer to the pressure sensor and therefore, attenuation occurred only if the left index finger received the touch impulse. In both of these cases, proprioception must report the proximity between the location of the press and the felt impulse. Thus, our study reveals that the proprioception of the resting finger determines sensory attenuation. Please note, that there was no explicit instruction to press the left index finger, but only to press the sensor. Future research might try to find out - in a virtual reality setup for instance - whether proprioception triggers attenuation even if a subject does not intend or even know that she is touching a part of her own body.

Our results put constraints on the models of sensory attenuation. For the comparator model, they suggest that the prediction of the sensory consequences of the action that is contained in the forward model must contain the precise landing point of the touching movement. It is known that efference copies transfer spatially specific knowledge about the goal of a movement. For saccades, for instance, it has been shown that in a double-step paradigm in the absence of visual information, the second saccade is precisely informed about the landing position of the first saccade (Zimmermann, Morrone, & Burr, 2015). This information likely stems from an efference copy signaling direction and amplitude of the primary movement.

The preactivation account receives support by findings of spatial selectivity. The ideomotor theory claims that movement planning consists in the predictive activation of the precise sensory consequences that would ensue the movement. Thus, our results are fully consistent with the preactivation account. The active inference theory is akin to the ideomotor theory in that it similarly claims that predictions (or better prediction errors) about the consequences of movements incite these movements. However, unlike the preactivation account, active inference does not count the tactile sensation on the touched finger to the direct movement consequences. The latter consist mainly of proprioceptive changes in the moving finger, according to active inference. As the aim of a movement - according to active inference - is to reduce prediction errors, proprioceptive changes must be particularly registered during movement execution. To highlight the comparison of proprioceptive predictions, the precision of sensory evidence will be reduced, thus decreasing the intensity of sensations in the remaining sensory channel. According to this theory, the reduction in precision is applied to the entire sensory channels during the movement. The advantage of this conception is that sensory attenuation for external events, like sounds produced by a button press, follow as a necessary consequence. However, this theory is not consistent with our results. We show that not the sensitivity of the entire channel is reduced, but only the precise region that is the goal location of the touching movement. Our experimental setup only allowed predictions based on motor, i.e. efference copy information.

## 5 Conclusion

In summary, our results show that sensory attenuation of self-touch is spatially specific, being restricted to the goal location of the touching movement only.

## Credit authorship contribution statement

**Franziska Knoetsch:** Data collection, Data analysis, Writing - Original draft preparation. **Eckart Zimmermann:** Software, Data analysis, Writing - Original draft preparation.

## References

[b0005] Baess P., Widmann A., Roye A., Schröger E., Jacobsen T. (2009). Attenuated human auditory middle latency response and evoked 40-Hz response to self-initiated sounds. European Journal of Neuroscience.

[b0010] Bays P.M., Wolpert D.M. (2008). Predictive attenuation in the perception of touch. Attention & Performance.

[b0015] Blakemore S., Wolpert D., Frith C. (2000). Why can't you tickle yourself?. Neuro Report.

[b0020] Blakemore S.J., Frith C.D., Wolpert D.M. (1999). Spatio-temporal prediction modulates the perception of self-produced stimuli. Journal of cognitive neuroscience.

[b0025] Blakemore S.J., Wolpert D.M., Frith C.D. (1998). Central cancellation of self-produced tickle sensation. Nature Neuroscience.

[b0030] Brown H., Adams R.A., Paress I., Edwards M., Friston K. (2013). Active inference, sensory attenuation and illusions. Cognitive Processing.

[b0035] Burin D., Pyasik M., Salatino A., Pia L. (2017). That's my hand! Therefore, that's my willed action: How body ownership acts upon conscious awareness of willed actions. Cognition.

[b0040] Cardoso-Leite P., Mamassian P., Schütz-Bosbach S., Waszak F. (2010). A new look at sensory attenuation. Action-effect anticipation affects sensitivity, not response bias. Psychological Science.

[b0045] Cicchini G.M., Anobile G., Burr D.C. (2014). Compressive mapping of number to space reflects dynamic encoding mechanisms, not static logarithmic transform. Proceedings of the National Academy of Sciences.

[b0050] Cicchini G.M., Mikellidou K., Burr D.C. (2018). The functional role of serial dependence. Proceedings of the Royal Society B.

[b0055] Cont C., Zimmermann E. (2021). The motor representation of sensory experience. Current Biology.

[b0060] Desantis A., Roussel C., Waszak F. (2014). The temporal dynamics of the perceptual consequences of action-effect prediction. Cognition.

[b0065] Dogge M., Custers R., Aarts H. (2019). Moving Forward: On the Limits of Motor-Based Forward Models. Trends in Cognitive Sciences.

[b0070] Dogge M., Hofman D., Custers R., Aarts H. (2019). Exploring the role of motor and non-motor predictive mechanisms in sensory attenuation: Perceptual and neurophysiological findings. Neuropsychologia.

[b0075] Fischer J., Whitney D. (2014). Serial dependence in visual perception. Nature Neuroscience.

[b0080] Fritsche M., Mostert P., de Lange F.P. (2017). Opposite effects of recent history on perception and decision. Current Biology.

[b0090] Hughes G., Desantis A., Waszak F. (2013). Mechanisms of intentional binding and sensory attenuation: The role of temporal prediction, temporal control, identity prediction, and motor prediction. Psychological Bulletin.

[b0095] Hughes G., Waszak F. (2011). ERP correlates of action effect predic- tion and visual sensory attenuation in voluntary action. NeuroImage.

[b0100] Kilteni K., Ehrsson H.H. (2017). Sensorimotor predictions and tool use: Hand-held tools attenuate self-touch. Cognition.

[b0105] Kilteni K., Engeler P., Ehrsson H.H. (2020). Efference copy is necessary for the attenuation of self-generated touch. IScience.

[b0110] Kilteni K., Houborg C., Ehrsson H.H. (2019). Rapid learning and unlearning of predicted sensory delays in self-generated touch. Elife.

[b0115] Mifsud N.G., Oestreich L.K., Jack B.N., Ford J.M., Roach B.J., Mathalon D.H., Whitford T.J. (2016). Self-initiated actions result in suppressed auditory but amplified visual evoked components in healthy participants. Psychophysiology.

[b0120] Roussel C., Hughes G., Waszak F. (2013). A preactivation account of sensory attenuation. Neuropsychologia.

[b0125] Schwarz K.A., Pfister R., Kluge M., Kunde W. (2018). Do we see it or not? Sensory attenuation in the visual domain. Journal of Experimental Psychology: General.

[b0135] Weiss C., Herwig A., Schütz-Bosbach S. (2011). The self in action effects: Selective attenuation of self-generated sounds. Cognition.

[b0145] Yon D., Press C. (2017). Predicted action consequences are perceptually facilitated before cancellation. Journal of Experimental Psychology: Human Perception and Performance.

[b0150] Zimmermann E., Morrone M.C., Burr D. (2015). Visual mislocalization during saccade sequences. Experimental Brain Research.

